# LeafSpec-Dicot: An Accurate and Portable Hyperspectral Imaging Device for Dicot Leaves

**DOI:** 10.3390/s23073687

**Published:** 2023-04-02

**Authors:** Xuan Li, Ziling Chen, Jialei Wang, Jian Jin

**Affiliations:** Department of Agricultural and Biological Engineering, Purdue University, West Lafayette, IN 47907, USA

**Keywords:** hyperspectral imaging device, automation, high resolution, nutrient detection, portable, dicot plants leaf-level proximal sensor

## Abstract

**Highlights:**

**What are the main findings?**

- The first portable hyperspectral imaging device specially designed for dicot plants to capture the image of an entire soybean leaf.
- The prediction of nitrogen content using images captured from the device establishes a strong correlation with the nitrogen content measured via chemical analysis.

**What is the implication of the main finding?**

- The imaging process is fully automated to maintain the consistency of images and relive the labors from operators.
- The device allows users to see the leaf more clearly which could open new pathways for plant study.

**Abstract:**

Soybean is one of the world’s most consumed crops. As the human population continuously increases, new phenotyping technology is needed to develop new soybean varieties with high-yield, stress-tolerant, and disease-tolerant traits. Hyperspectral imaging (HSI) is one of the most used technologies for phenotyping. The current HSI techniques with indoor imaging towers and unmanned aerial vehicles (UAVs) suffer from multiple major noise sources, such as changes in ambient lighting conditions, leaf slopes, and environmental conditions. To reduce the noise, a portable single-leaf high-resolution HSI imager named LeafSpec was developed. However, the original design does not work efficiently for the size and shape of dicot leaves, such as soybean leaves. In addition, there is a potential to make the dicot leaf scanning much faster and easier by automating the manual scan effort in the original design. Therefore, a renovated design of a LeafSpec with increased efficiency and imaging quality for dicot leaves is presented in this paper. The new design collects an image of a dicot leaf within 20 s. The data quality of this new device is validated by detecting the effect of nitrogen treatment on soybean plants. The improved spatial resolution allows users to utilize the Normalized Difference Vegetative Index (NDVI) spatial distribution heatmap of the entire leaf to predict the nitrogen content of a soybean plant. This preliminary NDVI distribution analysis result shows a strong correlation (R^2^ = 0.871) between the image collected by the device and the nitrogen content measured by a commercial laboratory. Therefore, it is concluded that the new LeafSpec-Dicot device can provide high-quality hyperspectral leaf images with high spatial resolution, high spectral resolution, and increased throughput for more accurate phenotyping. This enables phenotyping researchers to develop novel HSI image processing algorithms to utilize both spatial and spectral information to reveal more signals in soybean leaf images.

## 1. Introduction

The human population is expected to grow to over 9 billion by 2050 [1]. Because of the continuous increase in the human population and consumption of food per person, the global demand for food is expected to increase for the next 40 years [2]. However, the growth in agricultural production has not kept up with the population growth. Soybean is one of the most valuable crops in the world, as it is used for oil extraction, livestock feeds, and aquaculture. Soybeans are also a good source of protein and other nutrients for humans [3]. It is projected that the crop yield for soybeans will increase by 1.3% per year, which is not enough to accommodate the increase in demand [4]. To ensure food security for human society, high-yielding and stress-tolerant plants need to be selected and bred. Advanced techniques such as DNA sequencing allow plant scientists to breed plants molecularly [5]. However, the lack of access to phenotyping capabilities hinders plant scientists’ ability to relate genetics to plant growth, yield, and stress tolerance [1].

**Figure 1:**
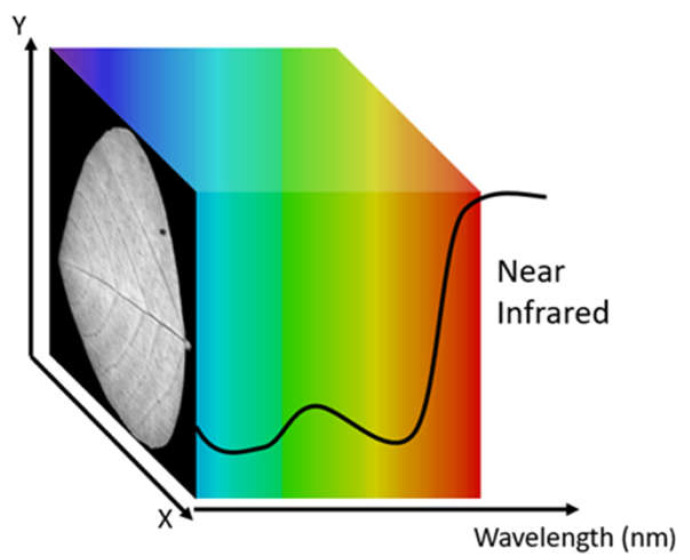
A visual illustration of a hyperspectral image cube (hypercube). The X- and Y-axes correspond to the image’s spatial width and height, respectively; the third dimension is the spectral dimension.

Hyperspectral imaging (HSI) can help plant scientists quantitatively choose the better breed of plants in a high-throughput and non-invasive manner [6]. In the traditional RGB camera, each pixel contains three values corresponding to the intensity of red, green, and blue. In contrast, a hyperspectral camera (HSC) can capture a spectrum for each pixel. The number of wavelengths in the spectrum can vary from tens of bands to hundreds of bands. The resulting dataset captured by the camera is called a hypercube, as illustrated in Figure 1. 

Hyperspectral imaging technologies have been applied in both greenhouses and fields. In greenhouses, the most popular method for collecting hyperspectral (HS) images was to enclose the entire plant inside an environmentally controlled box blocking all ambient light [7,8,9]. To increase the throughput of the imaging system, the entire process was automated by attaching the imaging system to a conveyer belt [10,11,12]. Another method for a high-throughput phenotyping system was to use an overhead crane system with an HSC mounted to capture top views of the plants [13,14].

All these HSI systems demonstrated their ability to predict nutrient content. However, there are three major sources of noise the systems could not avoid. The first noise source is the relative angle between the leaf and the HSC. When the leaf is perpendicular to the HSC, the reflectance of the leaf is at its maximum. The light intensity changes as the leaf angle deviates from the perpendicular position. If the same white reference is used to calibrate the HS images, the calibration will not be accurate for a leaf that is not perpendicular to the camera. The second noise source comes from specular reflection and shadows on the leaf. Specular reflection is a mirror-like surface reflection that can saturate the image and prevent the production of the desired spectrum. Similarly, shadows cause the spectrum to change as the incoming light source is not the same as the non-shadowed part of the plant. The last major noise source is the variance between the plant’s top and bottom leaves. The nutrient distribution throughout the plant is not uniform [15,16,17,18], which can overpower the signal of plant traits. The current method for processing the hyperspectral image is to average all the spectrums corresponding to the plant. Each plant is only represented by one spectrum, which means the signal between different plant sections is lost from the averaging operation.

In the field, an HSC has been installed on aerial (satellite or unmanned aerial vehicle (UAV)) and ground platforms. The typical use of an HSC on an aerial platform is on a UAV. There have been many UAV platforms designed to carry an HSC. The light-weight UAV developed at the University of Jyväskylä had an interferometer-based HSC. The data from the UAV were able to estimate the biomass and nitrogen of wheat fields. The estimated normalized root-mean-squared error (NRMSE) was 18.1% and 19.7% for biomass and nitrogen, respectively [19]. Multiple studies also correlated hyperspectral images with various traits [20,21,22]. HSCs have also been used on ground-based vehicles for various applications [23,24,25]. A team from The University of Sydney developed a ground base vehicle carrying an HSC to estimate mangoes’ maturity. The cross-validation regression result showed that the R^2^ value was 0.74 for the correlation between prediction and ground truth [26]. 

The current setup of the field HSI system uses sunlight as the light source for the camera. Depending on the time of the day and the location of the field, the sunlight intensity and angle can change drastically [27]. The normalized difference vegetation index (NDVI) of the plant follows a V-shaped pattern where the minimum is at solar noon time when the sun is at the highest location in the sky [28]. Additionally, a field hyperspectral camera can only achieve a 10 mm spatial resolution after orthorectification [29]. With a 10 mm spatial resolution, some of the pixels in the HS images will inevitably have the signal from plants and soil mixed, which can decrease the signal-to-noise ratio of the HS images. Although the current HSI systems, in the greenhouse or the field, have some noise sources that need to be reduced, the advantage of using such systems is that they have a very high throughput. In addition, the labor needed to gather the data is minimal as most of the system is either autonomous or semi-autonomous. 

To address the issues of current technologies, a handheld hyperspectral imaging device, LeafSpec, was developed for maize plants to take leaf-level HS images. LeafSpec enclosed the scanning section of the leaf into a small dark room. This setup allowed the lights and other environmental factors to be well controlled. Instead of the typical HSI setup with reflectance imaging, LeafSpec used transmittance imaging, providing better image quality by eliminating specular reflection [30]. The light source for the HSC was two halogen lights that provided a stable, smooth, and monotonic spectrum [31]. To further improve throughput, a robotic system was developed with a machine vision system to detect the target leaf and a cartesian robotic manipulator to grasp the corn leaf using LeafSpec [32].

However, the current configuration of LeafSpec is unsuitable for dicot leaves because of the morphological differences between soybean leaves and corn leaves. In addition, the current configuration requires human labor, which is not optimal for large-scale data collection. Moreover, the scanning quality across images is not consistent, due to manual scanning. 

Therefore, a new hyperspectral imaging device was designed to address these issues of current technology. The new design adopted automated systems to complete the scanning motion. The optical components, including the light source and path, were reconfigured to adapt to new configurations. After building the device, a set of experiments was carried out to validate the effectiveness of the device through correlating NDVI of the collected images with the nitrogen content of soybean plants.

## 2. Hardware Development

### 2.1. Overview

LeafSpec for soybean leaves is designed based on LeafSpec for corn leaves [30]. In the lightbox of LeafSpec for corn, there is a groove at the center to accommodate the midrib of corn leaves. The design also requires the LeafSpec device to slide across the entire length of the leaf. If one uses the same LeafSpec device on a soybean leaf, the dragging force would break the fragile leaf. 

**Figure 2:**
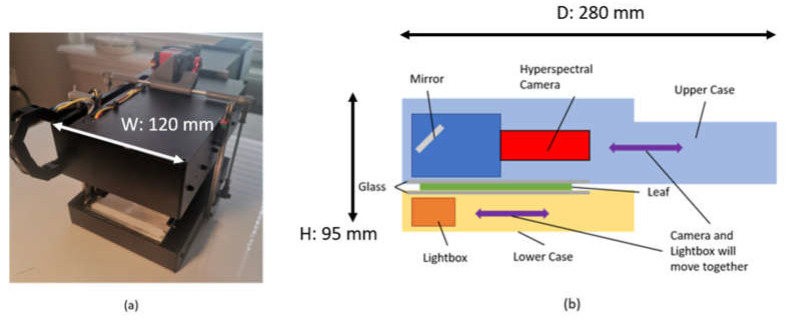
LeafSpec Handheld HSC for dicot leaf: (**a**) LeafSpec for dicot leaf picture; (**b**) Internal structure of the LeafSpec for dicot leaf device.

Therefore, LeafSpec for soybean leaves needs to avoid sliding across the leaf. The design of the LeafSpec for soybean leaves uses two sheets of glass to compress the leaf in between and protect it from sliding damage. The HSC and the lightbox are aligned on the opposite side of the glass sheets, with a motor driving the entire imaging setup across the leaf using a rack and pinion mechanism. The completed device is shown in Figure 2a and the internal structure of the LeafSpec device is shown in Figure 2b. This device is compact in size and weight to make it easier to carry between different locations. The exact dimension is 120 mm wide, 280 mm deep, and 95 mm high, and the device weighs 1.5 kg. A smartphone app can be used to receive previews of the HS images. The user can use the smartphone to command the LeafSpec device to save the full HS images and the metadata of the HS images.

### 2.2. Hardware Design

#### 2.2.1. Lightbox

One of the major noise sources for the current HSI system is the effect of lighting changes between images. To eliminate this noise, the LeafSpec device encloses the leaf in a dark chamber. The light source of the HSC is the lightbox on the opposite side of the leaf, which is installed with two 12 W halogen lights. Halogen lights are chosen because the light it emits covers a wide range of wavelengths from visible to infrared (IR) with a very smooth spectrum. As the HSC is a push broom (line scanning) camera, the lightbox is designed only to illuminate a narrow and long section of the leaf. A plastic sheet covers the outlet of the lightbox to diffuse light across the entire illuminating section. 

#### 2.2.2. Scanning Mechanism

The scanning mechanism consists of a rack and pinion, two sheets of glass, and a motor driver with an encoder. In the original LeafSpec device made for corn, the HSC was mounted vertically with respect to the leaf. If the current version of LeafSpec uses the same design, the total volume of the case required will be very large. Alternatively, a mirror is used to reflect the light 90 degrees so that the HSC can be placed parallel to the scanning bed. The leaf is pressed between two sheets of glass to protect the leaf from sliding damage from the HSC. The two sheets of glass flatten the leaf to eliminate the noise caused by the leaf slope. 

#### 2.2.3. Electronics

**Figure 3:**
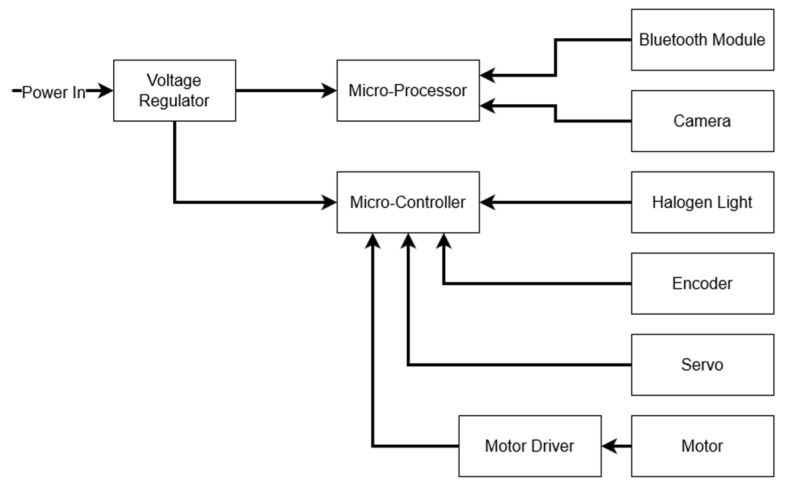
The electrical system of the LeafSpec consists of voltage regulators, microprocessors, microcontrollers, and peripherals for the microprocessor and microcontroller.

LeafSpec for soybean leaves has three sections in the electronic system: the power supply, microprocessor, and microcontroller. In Figure 3, the diagram shows how the entire electronic system connects.

##### Power Supply

The LeafSpec accepts 24 VDC as input voltage and reduces the current from the battery to the device. As the battery voltage changes over time, two voltage regulators are needed to provide steady voltage for the electrical systems of the LeafSpec device. One of the voltage regulators transforms the 24 VDC to 12 VDC for powering the halogen light. Another voltage regulator transforms the power from 24 VDC to 5 VDC to power the microprocessor, microcontroller, and peripherals. 

##### Microprocessor

The microprocessor of the LeafSpec acts as the brain of the entire operation. It handles the computation and storage of data, communication with a smartphone over Bluetooth, and sends commands to the microcontroller. This new version of LeafSpec uses a Raspberry Pi 4 as the microprocessor, and it processes the incoming image data from the camera into HS images. The Raspberry Pi has a Bluetooth module attached to send scan information and receive commands from a paired smartphone, which is the user interface of the LeafSpec device. The Raspberry Pi communicates with the microcontroller via a Universal Asynchronous Receiver-Transmitter (UART). 

##### Microcontroller

The microcontroller in the new version of LeafSpec actuates all the mechanisms and turns on/off the halogen lights. The LeafSpec uses an Arduino nano as the microcontroller. The Arduino controls the motor by using an H-bridge motor controller, which controls the speed and direction of the motor. Arduino also has hardware interruptions that are important for sensing encoder signals at high speed. On the micro-gear motor, there is a magnetic encoder that uses two hall-effect sensors. The encoder has a resolution of 12,000 counts per revolution, which means the encoder has a 0.004 mm special resolution. Two limit switches are connected to the Arduino to limit the range of the HSC scanning area. The halogen lights are controlled using a p-channel MOSFET (IRF520).

### 2.3. Device Operation and Data Flow

**Figure 4:**
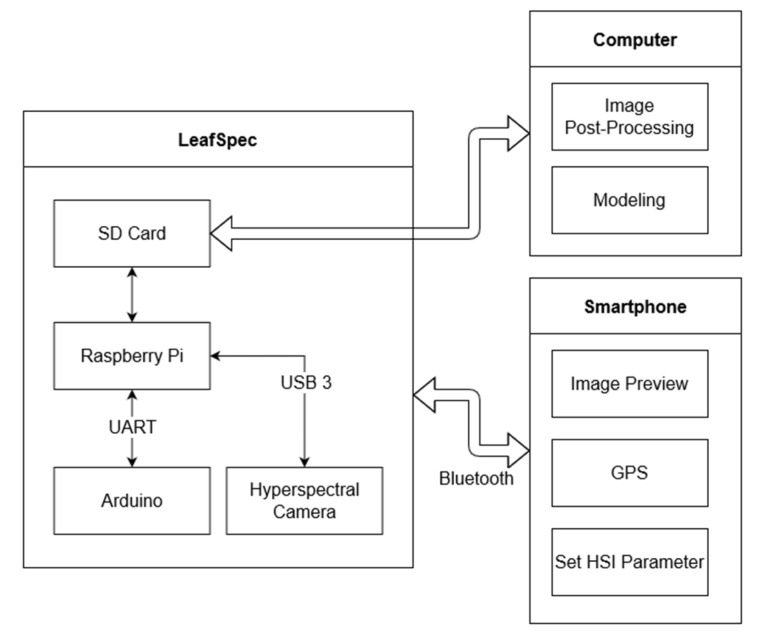
The data flow and management between the LeafSpec, smartphone, and computer.

An Android smartphone previews the HS images taken by the LeafSpec device and allows the user to input the metadata of the current HS images. The smartphone connects to the LeafSpec device using Bluetooth. Figure 4 shows how the data flows between different components of the LeafSpec system. 

The LeafSpec device can be triggered to start scanning by using a push-button for manual operation or a relay for autonomous operation. The Raspberry Pi initializes the HSC and sends a command to the Arduino to close the LeafSpec device and start spinning the motor. The motor runs continuously until reaching the limit switch. After the motor has started spinning, the Raspberry Pi starts acquiring images until the HSC has reached the end of the scanning bed. During imaging, the Raspberry Pi requests the current distance from the Arduino and then stores the image in RAM for later processing. Once the scanning has been completed, the Arduino moves the HSC back to its original position. The Raspberry Pi processes the data by organizing the images into a single hypercube. The Raspberry Pi also generates an NDVI heatmap for showing as a preview on the smartphone. The user can then save or discard the data on the smartphone. The full HS images’ hypercube data are saved on the SD card installed on the Raspberry Pi for further processing the HS images and modeling.

## 3. Validation of the Effectiveness of the Device through Correlating NDVI with Nitrogen Content of Soybean Plants

### 3.1. Overview

**Figure 5:**
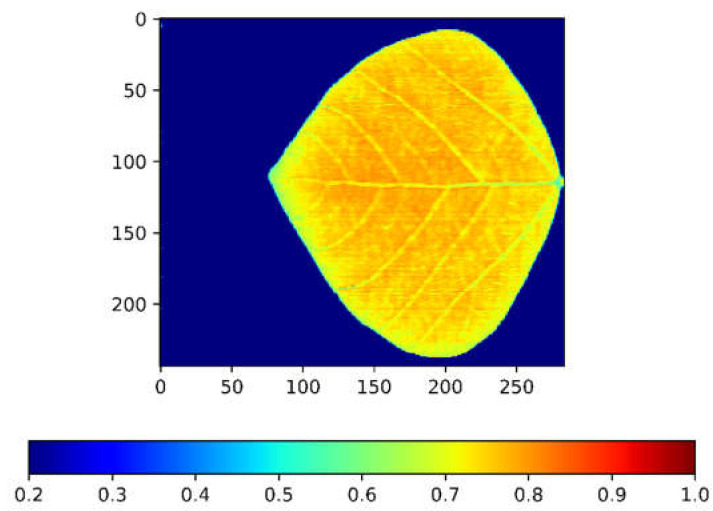
Whole-leaf NDVI heatmap captured by Leafspec-Dicot.

The Normalized Difference Vegetation Index (NDVI) is a powerful tool to investigate the nitrogen content of a plant [33,34]. The traditional method for understanding the nitrogen content of a leaf using HS images was by averaging the whole-leaf NDVI into one value. This method is useful for preliminary analysis as it can generate results easily and quickly. However, the mean NDVI is not capable of describing the distribution of the NDVI across the leaf, which could hinder the phenotyping results. For example, Figure 5 shows the whole-leaf NDVI heatmap of a soybean leaf taken by the Leafspec-Dicot device. Based on this image, the NDVI value at the edge of the leaf is lower than that at the center of the leaf, and the NDVI value at the veins is lower than that at the mesophyll. These distributions could be useful in analyzing specific plant phenomics but could not be captured by using the mean NDVI method. Therefore, by using Leafspec-Dicot to capture the whole-leaf NDVI heatmap, the correlation result should be improved when compared to the results of the mean NDVI method.

This study first developed a traditional linear regression model using the mean NDVI method to obtain a preliminary correlation result and establish a benchmark. Then, a deep learning neural network was used to analyze the whole-leaf NDVI heatmap taken by the Leafspec-Dicot device to investigate if the correlation can be improved. 

### 3.2. Data Collection

#### 3.2.1. Experimental Setup

The experiment for collecting HS images to train the nitrogen prediction model was conducted in the Purdue Lilly greenhouse in December 2020. A total of 64 soybean plants were grown under supplemental lighting for 10 h a day. The 64 plants were evenly split between two genotypes: Pioneer P34T21SE and Thorne. For each genotype, the 32 plants were split into four treatment groups: 8 plants with high-N treatment and well-watered, 8 plants with high-N treatment and drought-stressed, 8 plants with low-N treatment and well-watered, and 8 plants with low-N treatment and drought-stressed. The medium used in the experiment was a mix of 67% Metro Mix 510 (Sun Gro Horticulture) and 33% Greens Grade™ (Profile^®^) by volume. The N treatment was applied one week after transplanting the soybeans from germination plates to growing plots. The treatments were applied twice, one week apart. The concentration of the fertilizer used in the high-N-treatment plants was 200 ppm and that for the low-N-treatment group was 25 ppm. Initially, all plants were irrigated as needed. Four days before the sampling date, the irrigation for the drought-stressed plants was stopped.

#### 3.2.2. Collection of HS Images

The sampling date was chosen to be 17 December 2020, from 1:30 PM to 5:00 PM. For each plant, the top matured trifoliate was imaged by the new LeafSpec device as it has been shown that there are no significant differences in nitrogen content and relative water content (RWC) between the three leaves in a trifoliate (Samantha, 2020). Each leaf in the top matured trifoliate was imaged separately by cutting the leaves off the plant and scanned. In total, 192 HS images were collected from the experiment.

#### 3.2.3. Laboratory-Tested Nutrient Data Collection

To measure the N content, the entire plant from the soil and above was cut and placed in a paper bag. All the plants were then dried using a dryer until all the water evaporated. The dried plants were ground to fine powder and sent to A&L Great Lakes Laboratories for N content analysis. 

### 3.3. Data Analysis

#### 3.3.1. Pre-Processing and Modeling Setup

Before HS images taken by the LeafSpec can be used for modeling, white referencing calibration and leaf segmentation need to be completed. The raw HS images need white reference calibration to obtain the transmittance of the leaf because there are non-uniformities in the LeafSpec imaging system. First, the imaging sensor sensitivity is not uniform across all wavelengths. Second, the light source, which is the two halogen lights, does not have a uniform spectrum across wavelengths. Lastly, although a plastic sheet is used in the lightbox to diffuse the two halogen lights, there are still lighting variations across the width of the scanning line. The white reference calibration picture is taken at the beginning of the experiments by turning on the halogen light for two seconds and acquiring one image from the imaging sensor. The two-second wait time allows the spectrum from the halogen light to reach a steady state. For each line scanned by the HSC, a white reference calibration is needed. The transmittance can be calculated by dividing the raw HS images by the white reference pixel by pixel, as shown in Equation (1).

(1) $$transmittance=\frac{raw\;data}{white\;reference}$$

After the HS images were calibrated using white reference, the leaf pixels were segmented using the NDVI heatmap. The heatmap was calculated by applying the NDVI equation, Equation (2), to each spectrum in the HS images. The leaf could then be segmented using an NDVI threshold of 0.4.

(2) $$NDVI=\frac{NIR-RED}{NIR+RED}$$

where NIR is the intensity of the spectrum at 840 nm and RED is the intensity of the spectrum at 640 nm.

For the two methods of modeling that are discussed, the training dataset was the two of three leaves in the top matured trifoliate (128 HS images total). The testing dataset was the last leaf in the trifoliate (64 HS images total).

#### 3.3.2. Modeling Using Mean NDVI Method

**Figure 6:**
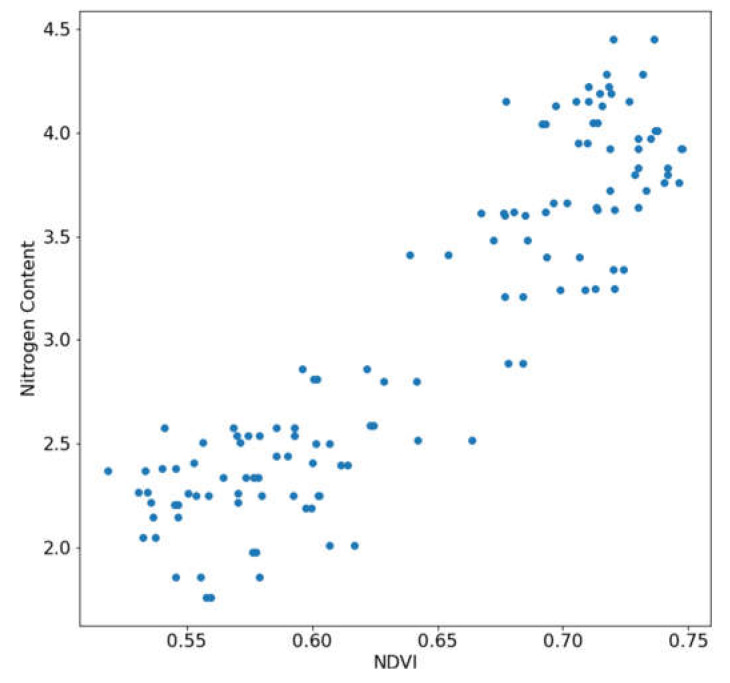
Linear relationship between nitrogen content and the mean NDVI.

The mean NDVI of the leaf was calculated using the segmented NDVI heatmap. By averaging all the NDVI values in the leaf pixels, one NDVI value represented one HS image. The mean NDVI value of each leaf against the N content ground truth was plotted, as shown in Figure 6. From the plot, a linear relation can be seen between the N content ground truth and the mean NDVI value of the leaf.

#### 3.3.3. Mean NDVI Method Correlation Result

A linear regression model was trained using the training dataset. The equation of the linear regression is:

(3) N content=9.897∗NDVI−3.316

**Figure 7:**
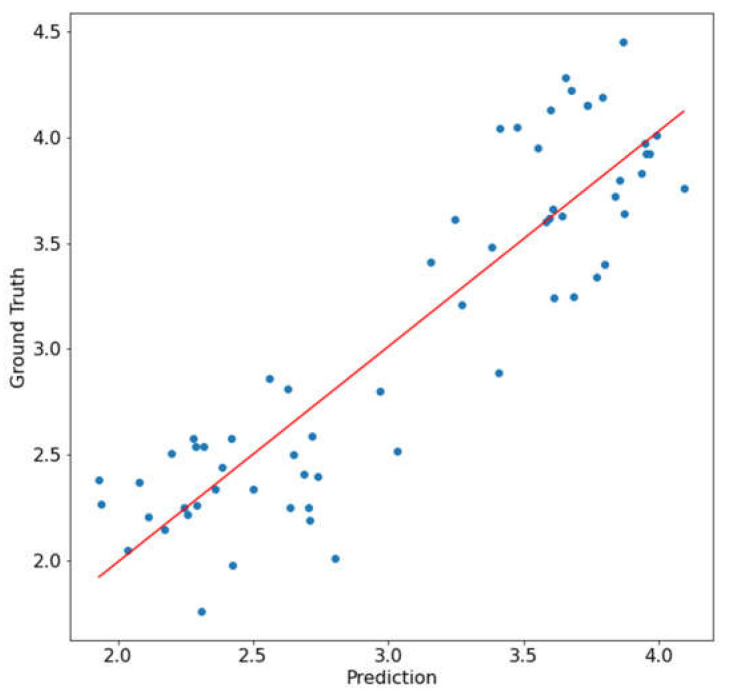
Correlation between N prediction and ground truth data using the mean NDVI modeling.

The linear regression model was then applied to the testing dataset and the correlation between the prediction and the ground truth data was plotted, as shown in Figure 7. The correlation had an R^2^ of 0.805 and a root-mean-squared error (RMSE) of 0.338. The strong correlation showed that the mean NDVI value of the leaf could be a good predictor for the N content of the entire plant.

#### 3.3.4. Modeling Using Whole-Leaf NDVI Heatmap

To utilize the NDVI distribution across the entire leaf, a deep learning neural network known as ResNet-34 was used to model the N content of the soybean plants. Compared with the traditional convolutional neural network (CNN), ResNet allows for a deeper network for the model to learn more complicated and abstract relationships within the training dataset. ResNet uses a residual network as the building block of the network and has been shown to be easier to optimize. ResNet can also achieve higher accuracy while keeping the number of parameters in the model low [35]. In the current application of N content prediction, a variant of ResNet, ResNet-34, was used. This network was chosen because it has the lowest number of parameters among all the variants. Keeping the number of parameters low is important because the training dataset is small.

The ResNet-34 was originally developed for image recognition for RGB images. The network needed a modification to read the NDVI heatmap, which was a grayscale image. The number of input channels of the first 2D convolution layer was changed to one for the grayscale input image. The output of ResNet was a tensor with a size of 1000. A fully connected layer was needed to reduce the tensor to the size of 1, which was the nitrogen content prediction. The loss function used for training is the mean squared error (MSE) loss, as shown in a general equation (Equation (4)), where *n* is the batch index, the input *x* is the nitrogen content measured from the laboratory, and the output *y* is the nitrogen content prediction made by the neural network based on the whole-leaf NDVI heatmap.

(4) $$l_{n}={\left(x_{n}-y_{n}\right)}^{2}$$

Before the training dataset was used to train the model, all the NDVI heatmaps were normalized to a mean of 0 and a standard deviation of 1. Normalization allows all the data to be on a common scale so that the model can be trained on the distribution within the image, not the differences in the range of values. 

#### 3.3.5. Whole-Leaf NDVI Heatmap Correlation Result

**Figure 8:**
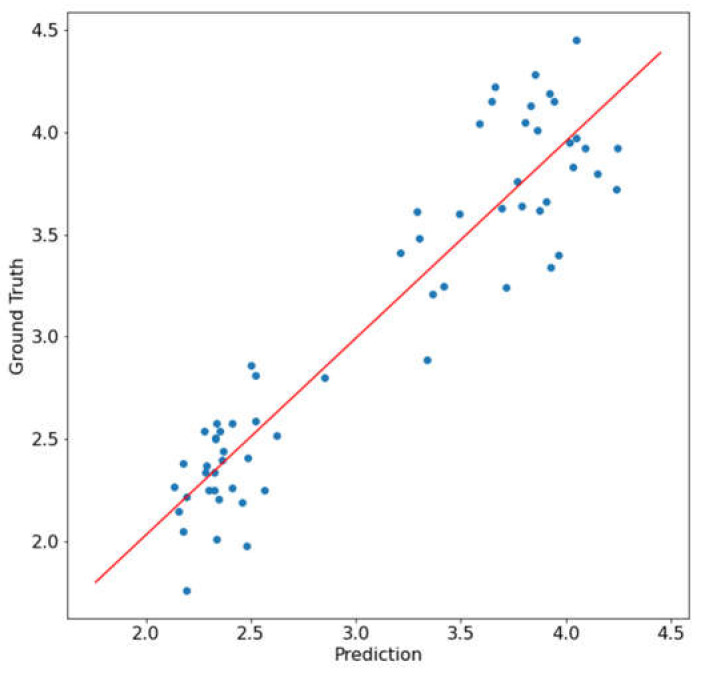
Correlation between N prediction and ground truth data using the NDVI heatmap distribution modeling.

The deep learning model was trained on the training dataset over 50 epochs and the model was applied to the test dataset. The correlation analysis was performed on the predicted N content. The scatter plot of the test dataset ground truth and prediction is shown in Figure 8. The results showed that the correlation between the ground truth and predicted N content had an R^2^ of 0.871 and an RMSE of 0.276. There was a significant improvement in the R^2^ value and the RMSE compared to the results from the mean NDVI method as the prediction based on the whole-leaf NDVI heatmap could use the distribution of the NDVI to improve the results. Moreover, the deep learning neural network could extract non-linear information from the whole-leaf NDVI heatmap that was not available in the mean NDVI method.

## 4. Conclusions

A new handheld hyperspectral leaf imager named LeafSpec-Dicot was developed to work with small and fragile soybean leaves with much faster scanning and higher imaging quality and resolution. The process of scanning the leaf using LeafSpec was automated by using a motorized rack and pinion mechanism. Through this study, it was demonstrated that using the whole-leaf NDVI heatmap on the leaf produced a strong correlation between the prediction and laboratory test results, which had an R^2^ of 0.871 and RMSE of 0.276, significantly improving the results obtained from the mean NDVI method (R^2^ = 0.805, RMSE = 0.338). As the Leafspec-Dicot can provide hyperspectral images with high spatial and spectral resolution, this device can be used for other applications such as studies about diseases and herbicides. We believe this innovation pushes the frontier of the current hyperspectral imaging device and makes this technology available to a larger population. 

## Data Availability

The authors would like to not share the data due to privacy.
